# Chronic arthritis leads to disturbances in the bone collagen network

**DOI:** 10.1186/ar2908

**Published:** 2010-01-15

**Authors:** Joana Caetano-Lopes, Ana M Nery, Helena Canhão, Joana Duarte, Rita Cascão, Ana Rodrigues, Inês P Perpétuo, Saba Abdulghani, Pedro M Amaral, Shimon Sakaguchi, Yrjö T Konttinen, Luís Graça, Maria F Vaz, João E Fonseca

**Affiliations:** 1 Rheumatology Research Unit, Instituto de Medicina Molecular, Faculdade de Medicina da Universidade de Lisboa, 1649-028 Lisbon, Portugal; 2 Instituto de Ciência e Engenharia de Materiais e Superfícies, Instituto Superior Técnico, Av. Rovisco Pais, 1049-001 Lisbon, Portugal; 3 Departamento de Engenharia de Materiais, Instituto Superior Técnico, Av. Rovisco Pais, 1049-001 Lisbon, Portugal; 4 Serviço de Reumatologia e Doenças Ósseas Metabólicas, Hospital de Santa Maria, 1649-035 Lisbon, Portugal; 5 Cellular Immunology Unit, Instituto de Medicina Molecular, Faculdade de Medicina, Universidade de Lisboa, 1649-028 Lisbon, Portugal; 6 Instituto Gulbenkian de Ciência, 2780-156 Oeiras, Portugal; 7 Department of Experimental Pathology, Institute for Frontier Medical Sciences, Kyoto University, Kyoto 606-8507, Japan; 8 University of Helsinki, Department of Medicine; ORTON Orthopaedic Hospital of the Invalid Foundation, 00281 Helsinki, Finland; 9 COXA Hospital for Joint Replacement, 33101 Tampere, Finland

## Abstract

**Introduction:**

In this study we used a mice model of chronic arthritis to evaluate if bone fragility induced by chronic inflammation is associated with an imbalance in bone turnover and also a disorganization of the bone type I collagen network.

**Methods:**

Serum, vertebrae and femur bones were collected from eight-month-old polyarthritis SKG mice and controls. Strength of the femoral bones was evaluated using three-point bending tests and density was assessed with a pycnometer. Bone turnover markers carboxy-terminal collagen cross-linking telopeptides (CTX-I) and amino-terminal propeptide of type I procollagen (PINP) were measured in serum. The organization and density of bone collagen were analyzed in vertebrae using second-harmonic generation (SHG) imaging with a two-photon microscope and trabecular bone microstructure was assessed by scanning electron microscope (SEM).

**Results:**

Femoral bones of SKG mice revealed increased fragility expressed by deterioration of mechanical properties, namely altered stiffness (*P *= 0.007) and reduced strength (*P *= 0.006), when compared to controls. Accordingly, inter-trabecular distance and trabecular thickness as observed by SEM were reduced in SKG mice. PINP was significantly higher in arthritic mice (9.18 ± 3.21 ng/ml) when compared to controls (1.71 ± 0.53 ng/ml, *P *< 0.001). Bone resorption marker CTX-I was 9.67 ± 3.18 ng/ml in arthritic SKG mice compared to 6.23 ± 4.11 ng/ml in controls (*P *= 0.176). The forward-to-backward signal ratio measured by SHG was higher in SKG animals, reflecting disorganized matrix and loose collagen structure, compared to controls.

**Conclusions:**

We have shown for the first time that chronic arthritis by itself impairs bone matrix architecture, probably due to disturbed bone remodeling and increased collagen turnover. This effect might predispose patients to bone fragility fractures.

## Introduction

Bone is a dynamic tissue composed mainly of a type I collagen matrix that constitutes the scaffold for calcium hydroxyapatite crystal deposition. Remodeling is a continuous process in which osteoclast-mediated bone resorption is coupled with osteoblast-mediated bone matrix production. Biochemical markers of bone turnover are produced during this process and released into circulation [1], providing a read-out of its pace and the balance between bone loss and formation [2]. More specifically, bone-resorbing osteoclasts secrete hydrochloric acid, followed by resorption of the demineralized organic type I collagen matrix by cathepsin K, leading to release of carboxy-terminal collagen cross-linking telopeptides (CTX-I) [1] as a marker for bone degradation. During the formation, phase type I collagen is synthesized *de novo*, and this leads to the release of the amino-terminal propeptide of type I procollagen (PINP) [3] as a marker of bone formation.

The mechanical and physical properties of bone depend on a hierarchical spatial arrangement of its collagen fibrils [4], which leads the deposition pattern of the extracellular hydroxyapatite crystals and consequently influences the overall bone strength. One of the consequences of the remodeling process is the alignment of collagen fibrils along the direction of the load in the mature lamellar bone, thus providing maximal strength to the structure [5]. Precisely due to this nanolevel architecture and the nonsymmetric protein arrangement in a crystalline array, collagen has the ability to generate a second-harmonic signal [6]. Second-harmonic generation (SHG) arises when the electric field of the exciting light is strong enough to deform a molecule. When a molecule, like collagen superhelix, is not symmetrical, the resulting anisotropy creates an oscillating field at twice the frequency, the so-called second harmonic. This method has an additional advantage of not dissipating energy into the sample, so that the sample retains its properties. As a matter of fact, due to its helical structure, SHG signal generated by type I collagen fibers is very strong and practically unaffected by most of the sample processing methods so that either unstained or stained routine histological sections can be subjected to this method. SHG microscopy is a powerful tool that has been developed to assess the bone collagen with a high spatial resolution [7] and has already been applied to mice tail tendons, wound repair, and metastatic growth. A correlation between the SHG intensity, fibrillar morphology, polarization anisotropy, and signal orientation with impaired mechanical strength has been reported in osteogenesis imperfecta [8].

In a recent paper, we used the SKG mouse model to demonstrate the weakening effect of chronic arthritis on the biomechanical properties of the affected vertebral and femoral skeleton in terms of stiffness, ductility, and ultimate (fracture) strength, besides the minimum effects observed on bone density [9]. We now hypothesize that this effect would be due, at least in part, to the inflammatory derangement of normal bone remodeling, leading to a higher bone turnover and consequently to a disorganized collagen type I matrix. To test this hypothesis, we have used a mice model of arthritis and assessed markers of bone collagen metabolism associated with modern SHG-assisted collagen architecture analysis, bone structure, and mechanical evaluation.

## Materials and methods

### SKG arthritis model and BALB/c controls

The SKG mice model of arthritis develops an erosive chronic polyarthritis that affects both large and small joints, is rheumatoid factor-positive, and presents some of the systemic features of rheumatoid arthritis (RA). The SKG mouse has a BALB/c background [10] with a recessive point mutation in the *zap*-70 gene, which encodes a protein that has an important role in T-cell signal transduction. As a result, this mutation leads to a positive selection of otherwise eliminated autoimmune T cells. SKG mice are genetically prone to develop chronic polyarthritis triggered by zymosan or other dectin-1 agonists [11].

Ten female SKG and ten female BALB/c mice were bred and maintained under specific pathogen-free conditions. All experiments were conducted according to the guidelines of the Animal User and Institutional Ethics Committee, and the study was approved by the local ethics committee. A single intraperitoneal injection of 2 mg of zymosan (Sigma-Aldrich, St. Louis, MO, USA) was administered at 2 months of age. Joint swelling was monitored by inspection as follows: 0, no joint swelling; 0.1, swelling of one finger joint; 0.5, mild swelling of wrist or ankle; and 1.0, severe swelling of wrist or ankle, with a range of the total score varying from 0 to 5 [10]. At 8 months, SKG and BALB/c mice were sacrificed and femoral bones as well as lumbar vertebrae were dissected free of soft tissues and stored at -20°C. Immediately before testing, the samples were defrosted at room temperature.

### Biochemical analysis of collagen turnover markers

Determination of bone turnover markers was performed on morning fasting samples that were obtained just before the sacrifice. Two biochemical markers were assayed by enzyme-linked immunosorbent assay (ELISA): CTX-I as a measurement of bone resorption and PINP for bone formation. Both protein serum concentrations were determined according to the guidelines of the manufacturer (Immunodiagnostic Systems, Boldon, Tyne and Wear, UK) by ELISA with sensitivities of 2.0 and 0.7 ng/mL, respectively.

### Density measurements

The densities of femoral bones were measured prior to mechanical testing with a water pycnometer. The pycnometer was filled with water, with corrections being made to the ambient temperature following the Archimedes' principle, as previously described [9].

### Mechanical testing

All mechanical tests were performed using a universal testing machine (Instron 5566™; Instron Corporation, Norwood, MA, USA) with a load cell of 500 N. The biomechanical parameters were displayed from stress-strain curves by Bluehill 2 Software (copyright 1997-2007; Instron Corporation) and analyzed using MatLab 7.1 software (R14 SP3, copyright 1984-2006; The MathWorks, Inc., Natick, MA, USA). The software has the ability to build stress-strain representations from load-displacement points once initial dimensions are provided for each specimen. Femoral bones were collected at the time of sacrifice and were submitted to three-point bending tests [12].

For these tests, the span between the outer loading points was 5 mm, with the load being applied to the center of the femoral shaft at a cross-head speed of 0.01 mm/s. The parameters analyzed from the stress-strain curves were Young's modulus, ultimate stress, ultimate strain, stiffness, and work/energy until maximum stress. When an increasing force is applied to the femoral shaft using a cross-head, it leads first to fully elastic deformation of the bone so that if the force is released the bone assumes its former shape. This property of the bone is assessed by its stiffness and elastic Young's modulus. The maximum stress still enabling such reversible changes is referred to as the yield point. When more stress is applied, bone tissue starts to undergo microfractures and truly irreversible plastic changes, which are characteristic of a ductile material, occur. The maximum stress the bone can tolerate without breakage reveals its ultimate strength, beyond which a macroscopic fracture soon follows. Stress-strain curves were obtained as previously described [9].

### Second-harmonic generation and two-photon excitation microscopy

The third lumbar (L3) vertebrae was decalcified, embedded in paraffin, cut to 7- μm sections using a microtome, and deparaffinized to be inspected in a Zeiss LSM510 META laser scanning microscope (Carl Zeiss, Jena, Germany) featuring a Coherent Mira 900 femtosecond multiphoton excitation laser (Coherent, Inc., Santa Clara, CA, USA). Multicolor nonlinear microscopy of collagen was done through SHG using two-photon excitation. The signal was acquired by two opposing detectors: (a) the META spectral detector that was configured for bandpass detection between 390 and 430 nm and that collected light from a Zeiss Fluar × 20/0.75 objective; this signal was associated with a green look up table (LUT) and is referred to as backward SHG; and (b) the non-descanned detector that collected light from a Zeiss 0.8 numerical aperture condenser and that was filtered by a 390- to 430-nm bandpass filter; this signal was associated with the blue LUT and is referred to as forward SHG. The backward SHG channel detects the backscattered SHG signal, and the forward SHG channel receives the photons that are transmitted through the sample. The wavelength of the laser was set to 820 nm. The method of Jefferies [13] was applied to all acquired images to quantify the amount of trabecular area occupied by collagen detected by the forward SHG channel and by collagen detected by the backward SHG channel [14].

Image analysis was done using National Institutes of Health (NIH) ImageJ software (Wayne Rasband, NIH, Bethesda, MD, USA) by applying a 40- to 255-cycle per second (cps) intensity threshold for each channel to cover only the extracellular collagen matrix. All of the trabecular area was covered using a lower 20- to 255-cps threshold.

The forward-to-backward signal ratio (the fraction of the percentage obtained in the forward SHG channel divided by the one obtained in the backward SHG channel) was determined. This ratio reflects the collagen matrix organization and density, and a higher ratio is associated with a less dense matrix [8].

### Scanning electron microscopy

The first lumbar vertebrae (L1) was inserted into an epoxidic and transparent resin and mounted in a mixture of *Résine Mecaprex MA2 *(04008) and *Durisseur pour résine Mecaprex MA2 *(Presi SA, Tavernolles, Brié-et-Angonnes, France) in 100:12 ratio. After 24 hours, the surfaces of the vertebrae samples were polished using grid papers with granulometries of 1,000, 800, 600, 320, and 200 μm. After deposition of a uniform and thin gold layer, the samples were observed with an electron beam energy of 25 keV in a scanning electron microscope (Hitachi model S2400 scanning electron microscope; Hitachi, Ltd., Tokyo, Japan).

The images obtained were analyzed with the software ImageJ (W. Rasband, NIH). In this case, the region of interest was the vertebral body, where pictures were taken at a magnification of × 100 and parameters such as the inter-trabecular distance (micrometers) and trabecular thickness (micrometers) were determined. These measurements were made according to the methods of Heyn and Jefferies [13].

### Statistical analysis

Results were presented as mean and standard deviation values. According to the distribution of the variables, either the *t *test for independent samples or the nonparametric Mann-Whitney test was used to compare continuous variables. Significance level was set at 0.05. Statistical analyses were performed using the Statistical Package for Social Sciences Manager software (SPSS, Inc., Chicago, IL, USA).

## Results

### Female SKG mice develop chronic arthritis

Ten female SKG mice injected with a single intraperitoneal injection of 2 mg of zymosan at 2 months of age developed chronic arthritis after 1 week (in both small and large joints), and the arthritis reached peak activity by the sixth week. During the first weeks after zymosan administration, scores were calculated every week and then every 2 weeks until sacrifice. By the time of sacrifice (8 months of age), the SKG mice had a disease duration of approximately 6 months and all presented a clinical activity score of 5, indicating persistent active disease. BALB/c mice were used as controls since they have the same genetic background as SKG and, consequently, the bone phenotype is common between these two strains before the development of arthritis. Control BALB/c mice (n = 10) did not develop arthritis (arthritis score = 0) after zymosan administration. At 8 months, SKG mice had a lower body weight than their BALB/c controls (18.9 ± 2.1 g versus 25.6 ± 1.8 g, *P *< 0.001).

### Bone collagen turnover is increased in chronic arthritis

PINP, the marker for bone formation, was higher in arthritic mice (9.18 ± 3.21 ng/mL) compared with controls (1.71 ± 0.53 ng/mL, *P *< 0.001). CTX-I, the serum marker for bone resorption, was also higher in arthritic mice (9.67 ± 3.18 ng/mL) compared with controls (6.23 ± 4.11 ng/mL) but without reaching statistical significance (*P *= 0.178).

### Chronic arthritis has reduced impact on bone density

The density measurements were lower in the arthritic group (1.26 ± 0.10 g/cm^3^) as compared with the control group (1.43 ± 0.23 g/cm^3^), although the results were not statistically different (*P *= 0.180).

### Chronic arthritis reduces mechanical properties of femurs

Mechanical three-point bending test results (Table 1) showed that arthritic femoral bones had a low elastic (Young's) modulus (reflecting reduced stiffness, *P *= 0.007) and ultimate stress strength (reflecting the maximum strength of the bone, measured at the point of fracture, *P *= 0.006) compared with control bones. The ultimate strain (ductility) and energy absorbed until ultimate point (toughness) did not differ.

**Table 1:** Values calculated from mechanical bending stress-strain curves of mice femur

	SKG	BALB/c	*P *value
Young's modulus, gigapascals	3.6 ± 0.8	6.1 ± 1.6	0.007
Ultimate stress, megapascals	123.7 ± 18.5	164.7 ± 22.5	0.006
Ultimate strain, percentage	6.9 ± 2.5	5.6 ± 1.5	0.280
Energy until ultimate point, Newton-millimeters per cubic millimeters	4.0 ± 1.9	4.7 ± 0.7	0.390

Values are presented as average ± standard deviation.

### Bone collagen structure is modified by chronic arthritis

**Figure 1:**
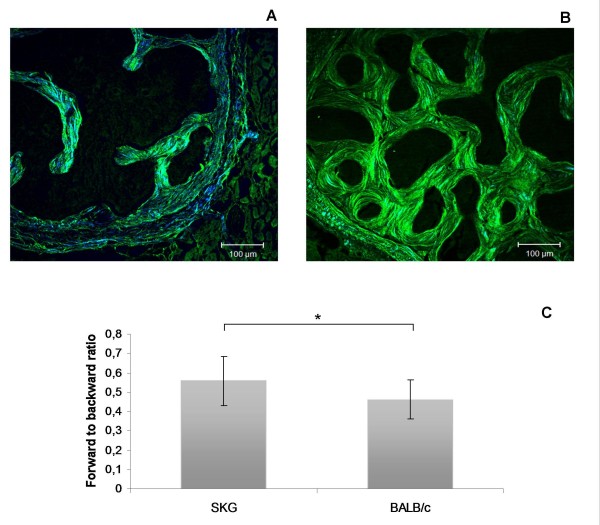
**Multiphoton microscopy images obtained from (a) SKG mouse vertebrae and (b) BALB/c mouse vertebrae**. The green color corresponds to backward SHG channel and the blue color to forward SHG channel. **(c) **The collagen organization, represented by the forward to backward ratio, was changed by arthritis. Scale bars = 100 μm. *P < 0.05. SHG, second-harmonic generation.

The percentage of collagen areas was calculated by analyzing the forward and the backward SHG channels separately. The values for the forward channel were 29.49% ± 7.36% in SKG mice and 23.19% ± 6.59% in controls. The corresponding values for the backward channels were 50.22% ± 9.70% and 53.85% ± 8.87%, respectively. The forward-to-backward signal ratio (the fraction of the percentage obtained in the forward SHG channel divided by the one obtained in the backward SHG channel) was higher in arthritic animals (0.566 ± 0.125) than in controls (0.453 ± 0.100, *P *= 0.005), reflecting a lower collagen matrix organization and density (Figure 1) [8].

### Vertebrae from arthritic mice have a higher inter-trabecular distance and a decreased trabecular thickness

In arthritic SKG mice vertebrae, the trabecular thickness was reduced, giving rise to an increased inter-trabecular distance in comparison with BALB/c mice vertebrae, as determined by scanning electron microscopy (Table 2).

**Table 2:** Trabecular measurements of L1 lumbar vertebrae, as determined by scanning electron microscopy

	SKG	BALB/c	*P *value
Trabecular thickness, micrometers	85.41 ± 43.54	99.12 ± 53.85	0.002
Inter-trabecular distance, micrometers	170.1 ± 74.9	142.2 ± 47.2	0.007

Values are presented as average ± standard deviation.

## Discussion

We have previously shown that arthritic mice bone has altered mechanical properties as compared with those from healthy mice [9]. We now show that this bone fragility is induced, at least in part, by chronic inflammation, leading to increased bone turnover that is coupled with impaired microarchitecture of the bone type I collagen network. In fact, we have documented that bone in arthritic animals has a higher remodeling rate, the increase in the bone-forming marker PINP being particularly evident, suggesting that an active bone-repairing process is ongoing. This may be due to inflammation-induced catabolism and bone destruction, which via coupling of bone resorption to bone formation lead to a compensatory osteoblast response in the form of increased bone matrix synthesis in an attempt to reinstall bone homeostasis. The question is whether this high rate of collagen production is able to fully compensate, both quantitatively and qualitatively, for the loss of bone organic matrix. It was therefore of interest to use the newly discovered potential of multiphoton microscopy to analyze this issue in more detail. Multiphoton microscopy in the forward and backward SHG mode was used to calculate the forward-to-backward signal ratio. This ratio, reflecting the bone collagen density and matrix organization [8], clearly showed that bone in the vertebrae of arthritic SKG mice had lower density and organization of collagen fibrils than the healthy control bone. An association between the SHG collagen pattern and mechanical strength was previously established in osteogenesis imperfecta [8], suggesting, together with our data, that this is a reliable method to assess bone collagen spatial organization.

Our results suggest that PINP is actually a more sensitive marker for increased bone turnover rate than CTX-I and this is quite in line with earlier studies of human RA. In fact, intervention studies with etidronate in RA suggest that the impact of the osteoclast-targeting treatment is actually more prominent on bone collagen type I synthesis than on collagen degradation markers [15]. The outcome of the collagen resorption versus synthesis observed by multiphoton microscopy analysis showed that there was a net loss of bone type I collagen in arthritis and that this is not due to diminished synthesis, as verified by the serum markers. Furthermore, SHG images clearly disclosed the disturbed and somewhat haphazard organization of the bone collagen fiber bundles in arthritic bone compared with the better organized lamellar architecture of the healthy control bone (Figure 1). Thus, it is concluded that in spite of the enhanced metabolic bone turnover rate in arthritic mice, this reparative attempt is able to maintain neither collagen mass nor its architectural quality (SHG signals assessed by multiphoton microscopy).

Collagen fiber orientation determines the topography and extent of the deposition of hydroxyapatite bone mineral in the extracellular bone matrix. Collagen further influences the elasticity of bone and is mainly responsible for its ability to absorb energy until the point of fracture, which can be assessed using the three-point bending test of arthritic and healthy control femoral bones. Indeed, it was shown that arthritic femoral bones had an impaired elasticity and relatively low ultimate (fracture) strength compared with healthy control femoral bones. This further supports our interpretation that the collagen component of arthritic bone is strongly affected by the inflammatory process and ultimately this interferes with bone mechanical performance. In addition, scanning electron microscopy findings showed that SKG mice exhibit thinner and more spaced trabeculae as compared with controls, suggesting the lack of a normal matrix-repairing process, which in this case is affecting the mineral deposition pattern and contributing directly to the low strength observed in the mechanical tests.

## Conclusions

In summary, we have concluded that the high remodeling rate of arthritic bone affects bone collagen fiber density and organization and leads to the deterioration of bone microstructure and mechanical properties.

## Abbreviations

cps: cycle per second; CTX-I: carboxy-terminal collagen cross-linking telopeptides; ELISA: enzyme-linked immunosorbent assay; LUT: look up table; NIH: National Institutes of Health; P1NP: amino-terminal propeptide of type I procollagen; RA: rheumatoid arthritis; SHG: second-harmonic generation.

## Competing interests

The authors declare that they have no competing interests.

## Authors' contributions

HC, YTK, and JEF helped to conceive the study and draft the manuscript. SS and LG helped to conceive the study. JD, RC, and IPP evaluated the mice and collected their biological material. AMN, MFV, PMA, and SA carried out the mechanical tests and scanning electron microscopy studies. JC-L did the immunoassays and the multiphoton microscopy, statistically analyzed the data, and helped to draft the manuscript. AR helped to draft the manuscript. All authors read and approved the final manuscript.
